# An Explanatory Model of Vascular Access Care Quality: Results of a Cross-Sectional Observational Study

**DOI:** 10.3390/nursrep14020079

**Published:** 2024-04-26

**Authors:** Sonia Casanova-Vivas, María Luisa Ballestar-Tarín, Pablo García-Molina, Ana Belén Lorente-Pomar, Ana Palau Gomar, Enrique Bdo. Hevilla Cucarella, José-María Blasco, Sonia Gomis-Baldoví

**Affiliations:** 1 Nursing Department, Facultat d’Infermeria i Podologia, Universitat de València, 46010 Valencia, Spain; sonia.casanova@uv.es (S.C.-V.);; 2 Servicio de Prevención de Riesgos Laborales, Conselleria de Sanidad, 46010 Valencia, Spain; 3 Nursing Care and Education Research Group (GRIECE), GIUV2019-456, Nursing Department, Universitat de Valencia, 46010 Valencia, Spain; 4 Hospital Arnau de Vilanova, Conselleria de Sanidad, 46015 Valencia, Spain; lorente_ana@gva.es; 5 Hospital Francesc de Borja de Gandia, Conselleria de Sanidad, 46702 Valencia, Spain; palau_anagom@gva.es; 6 Servicio de Análisis de Sistemas de Información Sanitaria, Conselleria de Sanidad Universal y Salud Pública, 46010 Valencia, Spain; hevilla_enr@gva.es; 7 Group of Physiotherapy in the Ageing Processes: Socio and Healthcare Strategies, Departamento de Fisioterapia, Universitat de València, 46010 Valencia, Spain; jose.maria.blasco@uv.es; 8 Hospital La Ribera, Conselleria de Sanidad, 46600 Valencia, Spain; gomis_sonbal@gva.es

**Keywords:** vascular access, intravenous therapy, quality, nursing care, indicators

## Abstract

The management of nursing care regarding patients’ vascular access is a priority. This study determines the contribution of the variables involved in the quality of care and maintenance of vascular access (VA) devices in admitted patients in the Valencian Community. Methods: Using the STROBE statement, an observational, cross-sectional study was conducted on 1576 VA devices. Data were collected using the INCATIV Questionnaire. We performed a multivariate analysis of the questionnaire variables. Results: In total, 50% had a good or very good assessment of the VA condition. This was positively correlated with anatomical location, dressing type, dressing date record, use of needle-free connectors (NFCs), date of last dressing change, presence of phlebitis, visibility of the insertion site and characteristics of the dressing’s condition (*p* < 0.001). The model indicated that the presence of phlebitis was the clearest predictor of a poor VA care assessment (OR = 20.579), followed by no visibility of the insertion site (OR = 14.209). Results also indicated that uncovered VA lumens or no NFCs used were related to a negative quality assessment. Conclusion: By managing and controlling these variables, the likelihood of providing optimal care is ensured. This enables the establishment of a standardised care approach for all nursing professionals and the building of a new quality indicator.

## 1. Introduction

In recent years, the management of nursing care regarding patients’ vascular capital has become a priority [1], which must always guarantee the safe insertion and handling of vascular access devices (VADs) [2]. Nevertheless, VADs are responsible for a large percentage of nosocomial infections, which have serious repercussions such as increased mortality rates, morbidity, days of admission, and healthcare expenditure [3]. According to the 2021 Study on the Prevalence of Nosocomial Infections in Spain (EPINE), 44.65% of the bacteraemia in Spanish hospitals is associated with a catheter. According to this same study, 12.34% of hospitalised patients have a central venous catheter (CVC), including peripherally inserted central catheters (PICCs), and 76.57% have a peripheral vascular catheter [4]. 

According to the guidelines of the Centers for Disease Control and Prevention (CDC) [5] or the RNAO Good Practice Guide for Vascular Access [2], for the prevention of complications associated with vascular accesses (VAs), the recommendation is to establish multidisciplinary strategies to improve compliance with evidence-based recommended practices. These include implementing a multi-component or multimodal care protocol, often referred to as a “care bundle”. A care bundle is a group of evidence-based interventions that can ensure the provision of a standardised care method, and in addition, several studies have already shown that its application can reduce complications [6,7,8].

The best way to evaluate the effectiveness of the proposed bundles is the use of indicators, as they allow relevant aspects of care to be objectified, comparisons to be made, objectives to be proposed, and a culture of the evaluation and improvement of care to be created [9].

The “Quality Indicators in Intravenous Therapy” (INCATIV) project developed an instrument for monitoring indicators to measure the quality of VA care in 2008 [10]. This project arose from the need to identify the state of nursing care for our patients’ VAs and to evaluate the impact of training and information interventions on the level of this care. The data collection instrument was the “INCATIV Questionnaire” which consisted of 22 variables related to VA, drawn up based on clinical guidelines and agreed upon by a group of experts with Delphi methodology. Based on this questionnaire, an indicator called the Standard Variable (SV-Gold standard) was used, which collected the main recommendations in care and measured the level of compliance with them. The results of this study concluded that training activities and monitoring improved the quality of VA care [10].

After evaluating this stage, and concluding that the SV was very demanding, the authors saw the importance of identifying which variables of the instrument had the greatest weight in influencing good VA care. Once these were identified, an indicator could be constructed and a bundle developed including all variables, which should become the main axis of the training of nursing professionals. 

To this end, this study aimed to determine the contribution of the variables involved in the quality of VA care in patients admitted to hospitals in the Valencian Community.

## 2. Materials and Methods

### 2.1. Design

An observational, analytical, cross-sectional study in five public hospitals in the Valencian Community was conducted in May 2016. Data collection was carried out by observers trained by the coordinating team using the INCATIV Questionnaire, already used in the previous stage of this study [10]. All hospital beds in all wards/units were checked. The inclusion criteria included any VA in patients admitted for more than 24 h. Exclusion criteria were the VAs of patients admitted to the Paediatric, Psychiatry, Emergency Room, Outpatient, and Dialysis units. This study was performed in accordance with the Strengthening the Reporting of Observational Studies in Epidemiology (STROBE) Checklist (Cross-sectional studies).

### 2.2. Data Collection Tool

The questionnaire consisted of 22 items divided into four parts in regard to patient-related variables, VA characteristics, daily VA care and complications (Table 1). Data collection was carried out by 15 nurses from the five participating hospitals, who were previously trained and approved for the use of the questionnaire by the coordinating group.

### 2.3. Statistical Analysis

A descriptive analysis of all variables was performed, showing the distribution of percentages for qualitative variables, and the mean and standard deviation for quantitative variables. The Chi-square test was used to study the relationships between the explanatory variables of the questionnaire (patient, VAD, and access condition variables, and related complications) with the response variable (VA condition assessment). 

Backward stepwise binary logistic regression was used to create an explanatory model of the condition of the VA (observer assessment) and the sociodemographic and access care variables. To construct this model, this variable was converted into a dichotomous one following a strict criterion, considering values 1, 2, and 3 (very bad, bad, or fair) as an incorrect assessment of the state of the VA, and values 4 and 5 (good and very good) as a correct assessment of VA care. The regression model included variables that were significant in the bivariate analysis using Chi-square with contrast statistics. The goodness-of-fit of the regression model was tested with the Hosmer–Lemeshow test. In all cases, statistical significance was set at *p* < 0.05. The predicted probability was obtained and contrasted with the response variable and the sensitivity and specificity of the model were calculated.

The statistical analysis was carried out with the SPSS 28.0 software package, licensed by the University of Valencia.

### 2.4. Ethical Considerations

The project complied with the ethical principles of all research. All the requirements set by the Department of Health of the Valencian Community were met. The study, being observational, did not involve intervention or changes in usual practice. We requested a waiver for patients’ informed consent as the study focused on assessing the quality of intravenous therapy and did not involve the collection of personal data. Being a multicentre study, approval was obtained from the Research Ethics Committee of the hospital (code PI070309), to which one of the coordinating group members belonged.

## 3. Results

During the study period, a total of 1576 VAs of patients admitted to the five participating hospitals were studied. Information was collected from all patients who met the inclusion criteria. Of the participants, 55.4% were men and 44.6% women. The distribution was similar in all the hospitals where data were collected. The mean age of the participants was 67.60 years (SD = 16.92). 

Table S1 (included in a supplementary file) presents the variables observed in the data gathered from the questionnaire. The data distribution per hospital is also included while maintaining their identification anonymous. The final variable included in the questionnaire is the healthcare professional’s assessment of the condition of the VA on a scale of 1 to 5, where 1 is very bad and 5 is very good (Table 2).

Access condition assessment was associated with the person’s age (*p* < 0.05). The mean age (Mean = 66.7, SD = 17.35) of those who received a good or very good VA assessment was lower than of those who did not receive an optimal VA assessment (Mean = 68.93, SD = 16.24) (*p* < 0.05). There were no differences in the VA condition assessment between men and women (χ^2^ = 0.957, *p* = 0.328). 

When relating the variables describing the VA with the evaluation of its condition, we observed that they were significantly related to the anatomical location of the VA (χ2 = 26.917, *p* < 0.001), the type of dressing (χ2 = 266.502, *p* < 0.001), dressing date record (χ2 = 44.352, *p* < 0.001), having the access uncovered (χ2 = 13.342, *p* < 0.001), the presence of a safety valve (χ2 = 17.338, *p* < 0.001), the date of last dressing change (χ2 = 27.894, *p* < 0.001), the presence of phlebitis (χ2 = 124.072, *p* < 0.001), the visibility of the insertion point (χ2 = 385.752, *p* < 0.001), and the different characteristics of the dressing’s condition, such as clean or dirty. These dressing characteristics were also significantly associated with the phlebitis variable.

The variables that were significant in the bivariate analyses were included in the regression model (Table 3); the variable associated with a better evaluation was used as a reference category. In this sense, it was noted that the anatomical location with the most favourable prognosis was the forearm. The type of dressing with the best result was the transparent one since it also allows for the insertion point to always be visible, unlike opaque dressings or so-called clumps (In Spanish, conglomerado o mazacote) (a term used to describe a dressing that does not allow for the insertion site to be visualised due to the recurrent use of gauze, bandages, or others). This surveillance also makes it possible to detect unwanted complications promptly, such as the presence of phlebitis. The condition assessment was also better when there was a record on the dressing of the date of insertion or care; and when a closed system was maintained, that was, there were no uncovered accesses and safety valves were used.

The summary of the estimated parameters for the main variables and the observer assessment in this model are shown in Table 4 and discussed below. The model has a good fit (X^2^ Hosmer-Lemeshow = 11.509, *p* = 0.174). The independent variables of the described regression model account for 47% of the variance of the dependent variable “Assessment of the state of vascular access care” (Cox & Snell R^2^: 0.350; Nagelkerke R^2^: 0.474), which can guarantee good quality and increase the possibilities of good VA care.

The model indicated that age influences VA care; the risk increases with age (OR = 1.008, 95% CI: 1.001–1.016). The anatomical location of the VA has an impact on VA complications; the risk is increased 4.5-fold if the access is located in the jugular (*p* < 0.001) and 1.9-fold if it is placed in the arm flexure (*p* < 0.001). The presence of an opaque or clump dressing increases the probability of a poor assessment of VA care by three to eight-fold (OR = 3.544 95% CI: 1.903–6.603; OR = 8.177, 95% CI: 3.536–18.912, respectively), as well as not recording the date of insertion on or in the vicinity of the dressing in short-term catheters, or the date of care in long-term catheters, has twice the risk of a poor evaluation (OR = 1.755, 95% CI: 1.388–2.301). 

The results also indicated that the evaluation is three times more likely to be negative if any of the VA lumens are uncovered (OR = 3.703; 95% CI: 1.918–7.151) or if these have no safety valves (NFC, needle-free connector) (OR = 1.537; 95% CI: 1.164–2.031). These items would reinforce the need to always use a closed system as a recommendation for the prevention of catheter-related infections.

The presence of phlebitis was the clearest predictor of a poor VA care assessment (OR = 20.579; 95% CI: 11.519–36.765). Furthermore, VAs in which the insertion point cannot be seen are 14 times more likely to have a bad assessment (OR = 14.209; 95% CI: 8.986–22.469).

The formula of the explanatory model of VA care was constituted as follows:

$$p=\frac{1}{1+exp(2.595-0.008X1-1.042X2-2.037X3-1.265X4-2.101X5-0.562X6-1.309X7-0.430X8-2.654X9-3.024X10)}$$

where:

*X*1 (age), *X*2 (anatomical location; flexure, where yes = 1 and no = 0), *X*3 (anatomical location; jugular, where yes = 1 and no = 0), *X*4 (opaque dressing, where yes = 1 and no = 0), *X*5 (clump dressing, where yes = 1 and no = 0), *X*6 (date record, where yes = 1 and no = 0), *X*7 (uncovered access, where yes = 1 and no = 0), *X*8 (no safety valve, where yes = 1 and no = 0), *X*9 (phlebitis, where yes = 1 and no = 0), *X*10 (visible insertion point, where yes = 1 and no = 0), and exponential value (2.595).

Based on the formula, the model calculates a predictive value between 0 and 1, where a value close to 0 indicates a non-optimal VA condition and 1 indicates a good VA condition. If we relate the professional’s evaluation (VA condition assessment) to our predicted value, it is significant (ρ = 0.471, *p* < 0.05). The indicators for assessing the discriminatory capacity of the explanatory model are shown in Table 5. We observed a sensitivity of 62.6 and a specificity of 93.1, and reported a positive predictive value of 85.4 and a negative predictive value of 79.4, with a validity index of 81.15. This demonstrates that the model has good discriminatory capacity.

## 4. Discussion

Adequate management of VA care should be achieved through the implementation of indicator-monitoring strategies, and training and evaluation measures [11]. This study has shown that the development of an explanatory model for the assessment of the quality of VA care allows for the identification of evidence-based practices on which training programs should focus. 

The application of bundles in the insertion and maintenance of central venous access devices (centrally inserted venous catheter, CICC; PICC; femoral-inserted venous catheter, FICC) has demonstrated, in different studies, a reduction in bacteraemia and other complications via continuous evaluation through indicators. Nonetheless, studies carried out applying bundles in the care and maintenance of peripheral VAs were of low quality and no conclusions could be reached as to their effectiveness [2,7,8].

According to the explanatory model of our study, the variables that influence the assessment of the VA condition are age, anatomical location, the presence of phlebitis, the type of dressing used, the date of VAD insertion on the dressing, the use of a closed system, and the visibility of the insertion site. 

No significant differences were found between males and females. In agreement with other studies, age does appear to be a risk factor; in a review study on good practices in peripheral catheter care, Zingg et al. [12] established that intrinsic factors such as age, disease severity, multiple comorbidities, or the length of hospital stay could increase the risk of infection. Other studies on the prevalence of difficult VAs also identified that age may be clinically relevant, given that elderly individuals undergo anatomical changes typical of ageing and tend to have a weaker vascular system, so extreme care should be taken in these patients [13].

In this study, the anatomical location chosen for VAD insertion indicates that it may have an impact on poor VA care if we choose the jugular for central access or the arm flexure for peripheral access. In different clinical practice guidelines (CDC [5], RNAO [2], SMP 2022 [4]), the choice of blood vessel for VAD insertion is identified as a risk factor for the development of associated bacteraemia; the order of risk ranging from the highest to the lowest is as follows: central venous (femoral, jugular, subclavian), pulmonary artery, peripheral venous, and peripheral arterial. A more recent study suggested that selecting the forearm is a protective factor for the cannulation of a difficult VA [14].

The importance of the visibility of the insertion site is another practice revealed by the explanatory model. Zingg [12] concluded in their latest study that if we cannot carry out a daily inspection of the insertion site, we will not be able to promptly detect some of the most frequent complications in any VAD, such as phlebitis, infiltration, mechanical failure, or displacement.

In our study, phlebitis was detected in 7% of cases. Being a cross-sectional study, we are dealing with low figures since this was the complication detected at that time. It is clear that, in this type of study, the presence of any symptom or sign of phlebitis is a clear indicator of poor VA care and should be corrected immediately. 

A study conducted by Milutinovic [15] identified the insertion site, catheter size, and duration as risk factors for phlebitis. Therefore, this could be prevented by correctly choosing the insertion site (hand/wrist on forearm), providing a good attachment, maintaining the appropriate duration, avoiding irritating infusions, checking the insertion site daily, and using a flexible polyurethane catheter (less thrombogenic, less rigid) [12]. To know the appropriate duration time, another variable explained by the model is the record of the VAD insertion date, which indicates this time. In this study, 35.5% of VADs did not comply with the guideline recommendations regarding date recording. Failure to document or visually represent this variable in the vicinity of the dressing indicates non-compliance with guideline recommendations [16,17,18,19].

For daily observation and the monitoring of the VAD insertion site, the use of an appropriate dressing and its maintenance are necessary. All current clinical practice guidelines agree that the appropriate dressing should be transparent, semi-permeable, and sterile. In this study, this was not used in almost 15% of cases. The model explains that the use of an opaque dressing or clump can increase the poor care of this VA by up to eight-fold. In addition, Rickard [20], in their SAVE-trial study, highlighted the importance of those aspects of the dressing related to cleanliness and comfort. The characteristics recommended based on greater scientific evidence are the comfort of the dressing and the patient’s perception, while those with less evidence being if the dressing is wet, dirty, or displaced [15]. The poor condition of the dressing can cause colonisation by microorganisms, which can lead to serious complications. For this reason, it is important to collect cleanliness and comfort characteristics, as well as the date of the last dressing change. 

Another variable revealed by the model is the recommendation to preferentially use NFCs in the access ports of venous catheters, as agreed in the previous good practice guidelines mentioned above. Finding an uncovered access or an area where NFCs are not used can signify a three-fold higher probability of a poor VA prognosis. 

The variables described by the model can constitute the minimum recommendations that a bundle should have. The application of these for both the insertion and maintenance of the central VAD has been shown, in different studies, to reduce bacteraemia and other complications if continuous evaluation is carried out using indicators; however, the studies applying bundles to the care and maintenance of peripheral VAs have been of low quality and no conclusions were reached as to their effectiveness [8].

To date, there is no study that identifies or quantifies the level of compliance with the practices included in the bundle for the care and maintenance of both central and peripheral VAs via a single nursing quality index. In health management, there are other indicators and scales that measure the frequency of adverse events or complications (falls, pressure ulcers, bacteraemia, etc.); however, none of them evaluate the compliance and quality level of nursing care in a practice as specific and frequent for nurses as VA care. 

This study’s strength lies in its comprehensive evaluation of both central and peripheral VAs across all participating units. The assessment was consistently conducted under the coordination of a team of experts, with the cooperation and support of hospital managers who facilitated its implementation. Training programs were conducted by personnel well-versed in the subject matter.

The study had limitations stemming from its observational nature, with bedside data collection from patients. This bias was reduced by homogeneous observer training. Some variables, including the type of medication, patient diagnosis, and specific antisepsis measures during both VAD insertion and maintenance, could not be collected, potentially impacting the assessment of care. Additionally, inherent to a cross-sectional study, the low occurrence of complications hindered the association of care and maintenance variables with adverse events.

## 5. Conclusions

This study has identified that age, anatomical location, the presence of phlebitis, the type of dressing used, the date of VAD insertion on the dressing, the use of a closed system, and the visibility of the insertion site are influential variables in assessing VA care for hospitalised patients in the Valencian Community. By managing and controlling these variables, the likelihood of providing optimal care is ensured. This enables the establishment of a standardised care approach for all nursing professionals.

This study reveals the need for further research to identify the predictors of the occurrence of complications and to study the impact of the training and implementation of new technologies that help achieve excellence in VA care.

## Figures and Tables

**Table 1:** Variables collected in the INCATIV questionnaire.

Patient-Related Variables	Variables Related to the Characteristics of the VAD	Variables Related to Daily VA Care	Vascular Access Complications
Age Sex Hospital of admission	Type of VAD VA Prescription VA Use Infusion type IV System type Anatomical location Calibre Number of lumens	Dressing type Dressing condition Dressing date recorded Last dressing change Insertion point visibility Post-catheter access 3-way stopcock Line condition	Presence of phlebitis and rating scale Access condition assessment

VAD, Vascular access device; VA, vascular access; IV, intravenous.

**Table 2:** Assessment of vascular access condition.

Assessment of VA Condition	N	%
Very bad	51	3.24
Bad	203	12.88
Fair	363	23.03
Good	610	38.71
Very Good	349	22.14

**Table 3:** Explanatory variables of the initial model.

Explanatory Variable	Categories
Age	Numeric variable
Location	1. Forearm (*Ref.) 2. Back of the hand 3. Wrist 4. Arm flexure 5. Upper third of the arm 6. Subclavian 7. Jugular 8. Reservoir
Dressing Type	1. Transparent (*Ref.) 2. Opaque 3. Clump 4. Transparent padded edges
Dressing Date Record	1. Yes (*Ref.) 2. No
Uncovered access	1. No (*Ref.) 2. Yes
Safety valve	1. No 2. Yes (*Ref.)
Insertion Point	1. Yes (*Ref.) 2. No

*Ref.: reference category in the regression analysis. The reference value is the one corresponding to an ideal response value.

**Table 4:** Variables included in the logistic regression model for the prediction of good VA care.

	B	Std. Error	Wald	Df	Sig.	Exp(B)	95% CI for EXP(B)	
							Upper	Lower
Age	0.008	0.004	4.355	1	0.037	1.008	1.001	1.016
VA Anatomical Location			30.224	7				
Forearm	0.405	0.179	5.108	1	<0.001			
Back of the hand	0.347	0.212	2.686	1	0.024	1.499	1.055	2.129
Wrist	0.679	0.185	13.400	1	0.101	1.415	0.934	2.142
Arm flexure	1.042	0.608	2.943	1	<0.001	1.971	1.371	2.834
Upper third of the arm	−0.569	0.518	1.203	1	0.086	2.835	0.862	9.328
Subclavian	1.517	0.394	14.787	1	0.273	0.566	0.205	1.564
Jugular	−2.037	1.585	1.652	1	<0.001	4.557	2.103	9.870
Reservoir	0.008	0.004	4.355	1	0.199	0.130	0.006	2.912
Dressing Type								
Transparent			36.732	3	<0.001			
Opaque	1.265	0.317	15.889	1	<0.001	3.544	1.903	6.603
Clump	2.101	0.428	24.130	1	<0.001	8.177	3.536	18.912
Transparent padded edges	0.052	0.237	0.049	1	0.825	1.054	0.663	1.676
There is no record of the dressing date	0.562	0.138	16.532	1	<0.001	1.755	1.338	2.301
Line Condition								
Access is uncovered	1.309	0.336	15.207	1	<0.001	3.703	1.918	7.151
There is no safety valve	0.430	0.142	9.161	1	0.002	1.537	1.164	2.031
Presence of phlebitis	3.024	0.296	104.346	1	<0.001	20.579	11.519	36.765
Insertion point is not visible	2.654	0.234	128.848	1	<0.001	14.209	8.986	22.469

B: beta; Std. error: Standard Error; Wald: Wald test; Df: Degrees of freedom; Sig: *p*-value <0.001 Exp(B): beta exponent; 95%CI: Confidence Interval. Model fit: X^2^ Hosmer-Lemeshow = 11.509, *p* = 0.174.

**Table 5:** Validity Indicators of the Explanatory Model of Vascular Access Care.

Validity Indicators	Value	95% CI	
		Lower Limit	Upper Limit
Sensitivity	62.6	58.74	66.38
Specificity	93.1	91.52	94.72
Positive Predictive Value	85.4	82.14	88.65
Negative Predictive Value	79.4	77.09	81.81
Validity Index	81.15	79.22	83.09

## Data Availability

Data are available upon reasonable request. All necessary data are supplied and available in the manuscript; however, the corresponding author will provide the dataset upon request. All data relevant to the study are included in the article.

## Supplementary Materials

The following supporting information can be downloaded at: https://www.mdpi.com/article/10.3390/nursrep14020079/s1, Table S1: Descriptive variables from the INCATIV Questionnaire.

## Public Involvement Statement

There was no public involvement in any aspect of this research.

## Guidelines and Standards Statement

This study was performed in accordance with the Strengthening the Reporting of Observational Studies in Epidemiology (STROBE) Checklist (Cross-sectional studies).
