# Supplementary material for: An Explanatory Model of Vascular Access Care Quality: Results of a Cross-Sectional Observational Study
Source: Nurs Rep. 2024 Apr 26;14(2):1049–57. doi: 10.3390/nursrep14020079 (PMC11130970; doi:10.3390/nursrep14020079)
Supplement: Supplementary file 1 [file nursrep-14-00079-s001.zip › nursrep-2853813-supplementary.pdf]

nursing reports

Table S1. Descriptive variables from the INCATIV Questionnaire.

	N	%
HOSPITALS		
H1	361	22,91
H2	113	7,17
H3	439	27,86
H4	315	19,99
H5	348	22,08
TYPE OF VAD		
PIVC (Peripheral IV catheter)	1410	89,5
CVC (Central IV Catheter)	91	5,8
PICC	44	2,8
Arterial Line	23	1,5
RSV (Reservoir, centra linel)	8	,5
VA PRESCRIPTION		
Fluid therapy	695	44,1
Medicación	531	33,7
Parenteral nutrition	26	1,6
None (possible non used line)	324	20,6
VA USE		
Continuous	700	44,4
Intermittent	816	51,8
In Y	60	3,8
INFUSION TYPE		
Gravity infusion	934	59,3
Pump	190	12,1
Infuser	19	1,2
Does not carry (intermittent line)	433	27,5
IV SYSTEM TYPE		
Closed	1257	79,8
Open	319	20,2
ANATOMICAL LOCATION		
Forearm	525	33,3
Back of hand	405	25,7
Wrist	224	14,2
Arm flexure	319	20,2
Upper third of the arm	14	,9
Subclavian	42	2,7
Jugular	38	2,4
Reservoir	9	,6
CANNULA SIZE PIVC		
18G	239	16,95
20G	661	46,88
22G	204	14,47
Others	306	21,70
CANNULA SIZE CVC		
4Fr	21	7,32
5Fr	47	16,38
6Fr	5	1,74
7Fr	122	42,51
8Fr	16	5,57
Others	76	26,48
NUMBER OF LUMENS		
One lumen	1453	92,2
Two lumens	47	3,0
Three lumens	76	4,8

DRESSING TYPE		
Transparent dressing	1226	77,8
Opaque dressing	119	7,6
Clumps ("Mazacote" inappropriate)	109	6,9
Transparent dressing padded edges	122	7,7
DRESSING DATE RECORDED		
Yes	1016	64,5
No	560	35,5
LAST DRESSING CHANGE		
0-3 days	1345	85,3
4-7 days	209	13,3
8, 9 o more days	22	1,4
INSERTION POINT VISIBILITY		
Yes	1286	81,6
No	290	18,4
POST-CATHETER ACCESS		
Bioconnector/safety shutter	64	4,1
Perforating membrane shutter	9	,6
3-way connector without extension	124	7,9
3-way without extension with bioconnect	82	5,2
Extension WITH 3-ay connector	365	23,2
Extension WITH 3-way WITH bioconnector	754	47,8
Bifurcated/trifurcated extension	51	3,2
Bifurcated/trifurcated extension with bio	127	8,1
THREE-WAY CONNECTOR		
One	1266	80,3
None	207	13,1
More than one	103	6,5
LINE CONDITION *(multiple selection)*		
Uncovered access	52	3,3
All accesses in use, connected	245	15,5
Connector cap	373	23,7
Perforating membrane shutter	55	3,5
Bioconnector	1067	67,7
Does not have line (Reservoir)	27	1,7
PHLEBITIS PRESENCE		
No	1458	92,5
Yes	118	7,5
